# Personality Traits and Risk of Eating Disorders in Men: A Cross-Sectional Study

**DOI:** 10.3390/healthcare11212910

**Published:** 2023-11-06

**Authors:** Rosendo Berengüí, María A. Castejón

**Affiliations:** Faculty of Education, Universidad Católica de Murcia (UCAM), 30107 Murcia, Spain

**Keywords:** eating disorders, personality traits, mental health, drive for thinness, bulimia, body dissatisfaction, neuroticism, extraversion, conscientiousness, openness to experience

## Abstract

Eating disorders (EDs) have been understudied and misunderstood in men. Among the relevant factors in the risk, onset, and maintenance of EDs, personality stands out. Therefore, the aim of the study was to analyze the relationships between personality traits and risk variables for the development of EDs in men. A total of 443 male university students (mean = 22.16 years) who completed the Spanish versions of the Eating Disorder Inventory-3 (EDI-3) and the NEO Five-Factor Inventory (NEO-FFI) participated. Correlation analyses were performed, and in order to determine the predictive role of personality traits on risk scales, a hierarchical multiple regression was performed. The results showed that neuroticism was positively associated with drive for thinness, being its main predictor variable. In bulimia, the main relationships were positively associated with neuroticism and negatively with conscientiousness. As for body dissatisfaction, the main predictor variables were neuroticism and, in a negative sense, extraversion and openness to experience. In conclusion, personality traits are related to the risk of developing EDs in male university students, with neuroticism being the main associated trait.

## 1. Introduction

Over the decades, it has been asserted that eating disorders (EDs) are diseases that primarily affect women, a notion that continues to be reinforced in current literature. Thus, the great majority of research on EDs, including symptom presentation, diagnosis, and treatment models, has been conducted using samples of women [1]. Studies have generally excluded men, which has increased the gap in male-focused knowledge, ED prevention, and specific clinical care [2,3].

However, the proportion of men with EDs and gender differences in body ideals, in addition to associated behaviors, may indicate that previous results of ED analysis, obtained primarily with women, may not be generalizable to men [4]. In fact, the estimated prevalence of EDs has ranged from 3.1% to 17.9% among females and 0.6% to 2.4% among males in the DSM-5 era [5]. Several studies indicate that women pursue a slim, underweight figure, while men seek a body ideal characterized by muscularity and thinness [6,7]. The related behaviors also tend to be different. While women are more likely to vomit and use laxatives to compensate for eating, men are more likely to over-exercise, abuse substances, restrict low-protein foods, prioritize the intake of more meals over other important tasks, or eat beyond satiety, among other behaviors [8,9,10]. In addition, it should be taken into account that, being considered a disease of women, men may believe that they do not suffer from EDs and because of the stigma they experience related to dealing with a psychological problem that typically affects women, they do not seek help [11,12].

Different biological, psychological, and social risk factors for the development of EDs have been confirmed in literature [13]. At the psychological level, different variables have been analyzed, which, in certain studies and samples, have been shown to be related to EDs, such as the drive for thinness, perfectionism, low self-esteem, body image problems, self-concept, deficient coping skills, and certain psychopathologies, such as anxiety, depression, and obsessive or compulsive disorders, among others [14,15].

Specifically in men, different risk factors, risk behaviors, and correlates have been studied. Body dissatisfaction is the most analyzed factor, with positive correlations between body dissatisfaction and the development of EDs [16,17], including higher symptomatology [18], binge eating, and subsequent purging [19] in people with higher dissatisfaction. They also tend to use unhealthy weight-control behaviors (e.g., increasing exercise, and dietary intake and skipping meals) [20], and compared to women, they manifest less concern about figure and weight, desire for thinness, and body dissatisfaction [21]. Depression, anxiety, obsessive–compulsive disorder (OCD), and specific mood disorders are conditions that can increase men’s risk of developing EDs [22]. Muscle dysmorphia has also been proposed as a risk factor for EDs in the male population [2], being associated with disordered eating and obsession with energetic intake [11,17].

Personality has also been a focus of interest in ED research. From the predispositional model, it is proposed that elevated levels of certain traits play a causal role in the development of EDs, increasing or decreasing the risk of EDs [23]. The association of personality traits with the risk of EDs has been confirmed in women [24,25,26,27], and so has its importance in clinical ED samples [23,26,28,29]. However, its analysis has been very limited in men.

Some of the findings in men point to relationships of neuroticism with an increased risk of EDs [15,25] and with disordered eating behavior [30], and high neuroticism and low conscientiousness were significant predictors of body dissatisfaction [31]. Similarly, higher scores on the trait responsibility were associated with a lower risk of ED [15].

Higher extraversion scores have also been found in men at low risk of ED, compared to men at medium or high risk, and negative correlations were found between extraversion and low self-esteem, personal alienation, interpersonal insecurity, interpersonal alienation, and emotional dysregulation [15].

For these reasons, EDs in men have come to be considered disorders that are underdiagnosed, undertreated and misunderstood [32], and early intervention is necessary to improve prevention in males; it is essential to understand how they occur and what their correlates are. The aim of this study was to analyze the relationships between personality traits and risk variables for the development of EDs in men.

## 2. Materials and Methods

### 2.1. Participants

A total of 443 adult men participated, with a mean age of 22.16 years (SD = 3.29) and an age range between 18 and 39 years. Initially, 455 students responded, although 12 were finally excluded due to errors and/or omissions in completing the instruments.

The criteria for inclusion in the study were to be of adult age and not to have been previously diagnosed with EDs or any other mental disorders.

All the participants were Spanish university students at public and private universities and from degrees of psychology (37.32%), education (29.09%), sports science (19.14%), and nursing (14.45%). Of the participants, 38.81% combined studies and work from different professions.

### 2.2. Instruments

Eating Disorder Inventory-3 (EDI-3). The EDI-3 inventory was used originally by Garner [33] and adapted to Spanish by Elosua et al. [34]. The inventory presents three specific scales of EDs, called Eating Disorders risk scales, specifically: (1) drive for thinness, consisting of seven items that assess the extreme desire to be thinner, preoccupation with food and weight, and an intense fear of gaining weight; (2) bulimia, consisting of eight items that assess predisposition to think about compulsive overeating and to compensate for binge eating by purging through vomiting; and (3) body dissatisfaction, with ten items that evaluate the dissatisfaction with the general shape of the body and rejection of the size of specific areas of the body. In this study, the reliabilities (Cronbach’s alpha) for the factors were: drive for thinness = 0.89, bulimia = 0.84, and body dissatisfaction = 0.83.

Five-Factor Inventory (NEO-FFI). To obtain the data on personality traits, the Spanish adaptation of the NEO-FFI was used [35], which is a reduced version of the Revised NEO Personality Inventory [36]. The instrument is composed of 60 items and allows the rapid assessment of the five major personality factors: (1) neuroticism (chronic predisposition to emotional distress versus emotional stability); (2) extraversion (energetic and thrill-seeking versus sober and solitary); (3) openness to experience (curious and unconventional versus traditional and pragmatic); (4) agreeableness (kind and trusting versus competitive and arrogant); and (5) conscientiousness (disciplined and meticulous versus laidback and careless). The NEO-FFI, both in its original version and in its Spanish adaptation, presents adequate psychometric properties and a good internal consistency in all dimensions. In this study, there was a good reliability (Cronbach’s alpha) for all factors: neuroticism = 0.85, extraversion = 0.80, openness to experience = 0.76, agreeableness = 0.75, and conscientiousness = 0.87.

### 2.3. Procedure

The sampling was non-random and by convenience.

Those responsible for the universities and degree programs were contacted in order to explain the objectives of the study and to request permission for data collection. Once approval was obtained, we proceeded to contact the different collaborating professors to request their collaboration in the application of the tests.

The questionnaires were answered collectively in the classroom after explaining to the participants the objectives of the study, the relevance of their participation, and the confidential treatment that the data obtained would receive.

The participation of the students was voluntary, and they could withdraw at any time; there was no compensation of any kind for their participation. In order to complete the questionnaires, it was necessary for the students to sign a consent form to participate in the study. The researchers were present during the application of the tests, supervising the correct filling out of the data and resolving any possible doubts that might arise.

### 2.4. Statistical Analysis

Data analysis was performed using the program IBM SPSS Statistics v.27 (IBM, Armonk, NY, USA). Descriptive statistics were calculated for continuous variables (means and standard deviations) and the reliability of the scales with Cronbach’s alpha (*α*). To perform the regression analyses, the normal distribution of the data was tested by skewness and kurtosis. We calculated Pearson’s correlation coefficients to observe patterns of common variation among the variables. In order to determine the predictive roles of personality traits on risk scales, a hierarchical multiple regression was performed. The significance level was set at a value of *p* < 0.05.

## 3. Results

Table 1 shows the descriptions of the scales evaluated and the correlations between them.

Drive for thinness showed a statistically significant correlation with neuroticism (*r* = 0.348) and a negative correlation with extraversion (*r* = −0.141). Bulimia was positively associated with neuroticism (*r* = 0.327) and negatively associated with extraversion (*r* = −0.139) and conscientiousness (*r* = −0.316). Body dissatisfaction was positively associated with neuroticism (*r* = 0.354) and negatively with all other traits: extraversion (*r* = −0.280), openness to experience (*r* = −0.262), agreeableness (*r* = −0.209), and conscientiousness (*r* = −0.196).

To proceed with the regression analyses, the normal distribution of the variables was assessed. Skewness (range −0.95 to 1.00) and kurtosis (range −0.50 to 1.20) values showed that the variables could be considered as having a normal distribution. In addition, the acceptable values in the variance inflation factor (VIF) and Durbin–Watson indicated that there were no multicollinearity and autocorrelation.

Following that, hierarchical regression analyses were performed. In Table 2, the predictor model was able to account for 12% of the variance in the drive for thinness. It can be seen that neuroticism was the only significant factor (*F*_1, 441_ = 60.812, *p* < 0.001).

Regarding the predictive role of personality traits on bulimia (Table 3), neuroticism entered the equation first, accounting for 11% of the model (*R*^2^ = 0.112, adjusted *R*^2^ = 0.110). Then, conscientiousness entered on the third step, accounting for an additional 6.1% of the variance (Δ*R*^2^ = 0.061). In the last step, neuroticism (*β* = 0.30, *p* < 0.001) significantly positively predicted bulimia, and conscientiousness (*β* = −0.30, *p* < 0.01) significantly negatively predicted bulimia.

In our last analysis, neuroticism explained 12% of the variance in body dissatisfaction (Table 4). In the next model, extraversion accounted for an additional 3% of the variance (*ΔR*^2^ = 0.029). It was observed, as in models 4 and 5, that when entering openness to experience, that factor accounted for an additional 6.4% of the variance (Δ*R*^2^ = 0.064). In the last model (*F*_5, 437_ = 20.375, *p* < 0.001), neuroticism (*β* = 0.32, *p* < 0.001), extraversion (*β* = −0.12, *p* < 0.001), and openness to experience (*β* = −0.17, *p* < 0.001) in the negative direction significantly predicted body dissatisfaction.

## 4. Discussion

The study of eating disorders among men has been, for several decades, a rarely analyzed topic, as it has been considered a problem that exclusively affects women. Even in the DSM classification of mental disorders, amenorrhea was one of the criteria for anorexia nervosa, something that remained until 2013, when it was eliminated [7,37]. The need to deepen the understanding of correlates and risk factors in male populations makes it necessary to continue proposing studies in this direction.

This study analyzed the relationships between personality traits and specific risk variables for the development of EDs in male university students. From Garner’s extended model [33], three main ED risk scales were proposed. The results of our study showed significant relationships between these risk scales and certain traits. Neuroticism was directly associated with the drive for thinness, being its main predictor variable. In bulimia, the main relationships were associated directly with neuroticism and negatively with conscientiousness. Regarding body dissatisfaction, the main predictor variables were neuroticism and, inversely, extraversion and openness to experience.

As has been found, the main personality trait associated with all risk scales is neuroticism. Neuroticism (vs. emotional stability) usually presents characteristics such as anxiety, hostility, depression, self-consciousness, impulsivity, or vulnerability, all of which are related to negative affectivity [36]. In several studies with general samples and with exclusively women, neuroticism was confirmed as a predisposing factor for EDs [24,38,39,40] and a key factor in the initiation, expression, and maintenance of eating disorders [41]. Additionally, it has been associated with the severity of ED symptoms in clinical and non-clinical populations [42,43,44]. Moreover, closely related to neuroticism, EDs are often comorbid with anxiety and depression disorders [15,45,46,47,48].

In the limited existing studies in men, high correlations were found between neuroticism and higher risks of EDs [15,25], with it being associated with higher-disordered eating behaviors [30].

In the results found, neuroticism is related to the drive for thinness and is its only significant predictor. Drive for thinness is one of the fundamental characteristics of EDs, which has been considered an essential criterion for diagnosis, according to different classification systems [49]. The EDI-3 scale assesses the extreme desire to be thinner, preoccupation with eating, preoccupation with weight, and intense fear of weight gain [33]. Drive for thinness is associated with negative consequences, such as dysfunctional and weight-regulation behaviors and clinically significant symptomatology [50].

In the past, it has been stated that the drive for thinness is associated, to a greater extent, with women [51] and the drive for muscularity with men [51,52]. Sociocultural norms in industrialized countries have suggested that a muscular physique is associated with masculinity [53]. However, both the drive for thinness and the drive for muscularity emerge in both sexes, and they may occur concomitantly [54]. Findings have also been found that provide support for the drive for thinness as an alternative transdiagnostic severity category for EDs in males, which may be more meaningful than the DSM-5 severity indices for anorexia nervosa and bulimia nervosa but not for binge eating disorder [55]. Therefore, due to the marked tendency to experience negative emotions and dissatisfaction, neuroticism may be related to a negative body image [56] and to the desire of men to maintain physical shape and not gain weight.

With regards to bulimia, we found that it is significantly related to neuroticism and negatively to conscientiousness. Bulimia in the EDI-3 is conceptualized as the tendency to suffer binge eating or uncontrolled bouts of food intake, most often in response to unpleasant emotional states [34]. In fact, people with high neuroticism suffer frequent mood changes and have a tendency toward emotional hypersensitivity, finding it difficult to return to normal after intense emotional experiences [36]. It is comprehensible that those people high in neuroticism present higher levels of bulimia and risks of EDs, due to the cited lack of control in food intake as a consequence of their negative emotional experience.

Previous studies with non-clinical populations have found high correlations of neuroticism with bulimia and preoccupation with food in females [57], high correlations of bulimia and a drive for thinness in males and females [15,42], and increased disordered eating behaviors, also for both males and females [26].

Regarding conscientiousness, it refers to the individual’s tendency toward organization and achievement, with the person being responsible, hardworking, competent, and organized, as opposed to people with low responsibility, who tend to have low self-discipline [58]. The negative relationship with bulimia seems to indicate that facets, such as organization, self-control, or scrupulousness, that characterize people with high responsibility [35] may constitute characteristics that protect against this lack of control in ingestion. A previous study found similar results to the present one, with a significant relationship between bulimia and conscientiousness, and higher levels of conscientiousness were associated with a lower risk of EDs only in men but not in women [15]. In addition, lower responsibility scores were found in ED patients than in control groups [59,60,61], and in women with EDs with a restrictive profile, a more responsible profile appeared than in patients who showed a compulsive/purgative eating profile [58]. It has also been suggested that low scores on conscientiousness and agreeableness and high scores on neuroticism and openness also increase ED risk [62].

Regarding body dissatisfaction, its main correlation was with neuroticism, and it was negatively related to the rest of the factors. Neuroticism, extraversion, and openness to experience were its predictors.

Body dissatisfaction expresses discontent with body shape and the size of certain body parts and is a risk factor responsible for the initiation and maintenance of external weight-control behaviors, leading to the development of EDs in those people who are vulnerable to them [33,34]. As in women, in men, body dissatisfaction is an essential element and one of the strongest risk factors that can lead to disordered eating and EDs [16]; the positive correlation between dissatisfaction and the development of EDs has been confirmed [2], and it is also associated with the increased symptomatology of EDs in men [18].

A negative perception may involve a lack of acceptance, leading to adverse emotional states, excessive weight control, a tendency towards perfectionism, and the internalization of standards of attractiveness [63]. Due to the tendency to experience negative emotions and dissatisfaction, neuroticism may be related to a negative body image [56]. Previous studies found high correlations of neuroticism with body dissatisfaction [15], which was associated with perceiving one’s own body as larger than it is objectively over time [64]. Additionally, neuroticism was a significant predictor and predicted dissatisfaction in both men and women [31]. This is related to the discrepancy between actual and ideal weights [56] and was useful in explaining emotions related to depression caused by discrepancies between ideal and self-weights [65].

With regards to extraversion, it defines the amount and intensity of interpersonal interactions, level of activity, need for stimulation, and capacity for joy [33]. The results suggest that it is a positive factor against body dissatisfaction, as it brings together characteristics such as sociability, activity, optimism, or affection.

The extraversion trait has gathered heterogeneous research results. On the one hand, in men, certain studies have found no association between extraversion and EDs and an increased risk of EDs [25,66]. On the other hand, the inverse association between extraversion, bulimic symptomatology, and body dissatisfaction has been confirmed [67], with higher extraversion scores in subjects with a low ED risk level [15]; additionally, lower extraversion has been related to EDs in women with high neuroticism [42]. Additionally, in previous studies, patients with EDs or subjects at a higher risk obtained lower scores in extraversion [60,68].

Finally, another trait that is negatively related to body dissatisfaction is openness to experience, which is characterized by breadth, depth and permeability of awareness, and active motivation to broaden and examine experience [33]. Like extraversion, facets such as creativity, imagination, or open-mindedness may be protective against body dissatisfaction.

This trait has reported the most contradictory results reported in literature, and there is little evidence of the role of this factor in relation to EDs or increased risk. The few existing studies showing evidence confirmed high levels of openness in EDs [59] and, conversely, lower openness in ED patients, with respect to control groups [60,62]. Moreover, higher scores for openness were related to a greater number of ED-related behaviors [69], and in patients with bulimic eating disorders, the remission and overall symptom reduction were positively predicted by this trait [70].

The study has several limitations. Firstly, one of the limitations is the nature of its design, since being a cross-sectional study only allows the variables to be analyzed at a specific moment in time; their evolution over time was not able to be verified. Therefore, the generalizability of the results is limited. The external transferability/validity of the results is also limited, with respect to other contexts, due to the fact that the people analyzed were university students and a proportion of workers. Additionally, another limitation related to this one is that the majority of the participants were psychology students, which represents an additional possible source of bias.

## 5. Conclusions and Future Perspectives

Personality is related to the risk of developing EDs in male university students. Trait neuroticism is related to the drive for thinness, bulimia, and body dissatisfaction risk scales, being the main predictor of the variance in all three. In addition, together with neuroticism, trait conscientiousness is a significant predictor of bulimia, with extraversion and openness to experience being negatively related to body dissatisfaction.

The study of correlates of EDs, such as personality, is necessary, especially in men. Since personality traits play an important role in the etiology, symptomatology, and maintenance of disordered eating behaviors in EDs [28,41], further personality analysis and surveillance of the most vulnerable subjects are required.

Men are a particularly relevant group in this area [71]. Currently, in prevention and treatment settings, men at risk or with EDs may delay seeking help or treatment to avoid the feeling of being “the odd one out” [72] and thus may seek help from services later in the disease, when symptoms may be more severe [11,12].

In addition, health promotion, early intervention, and the detection of vulnerable and at-risk individuals should be addressed from an early age, as it has been suggested that eating and body disorders in adolescence may be an important marker for the development of mental health difficulties later in life [73].

Finally, longitudinal studies are needed to help determine how personality traits may affect EDs in order to establish the direction of causality and to clarify the roles of traits as predictors of EDs.

## Figures and Tables

**Table 1:** Bivariate correlations between risk scales, psychological scales, and personality and descriptive statistics.

	1	2	3	4	5	6	7	8
1. Drive for thinness	1							
2. Bulimia	0.446 **	1						
3. Body dissatisfaction	0.654 **	0.432 **	1					
4. Neuroticism	0.348 **	0.327 **	0.354 **	1				
5. Extraversion	−0.141 **	−0.139 **	−0.280 **	−0.348 **	1			
6. Conscientiousness	−0.045	−0.316 **	−0.262 **	−0.261 **	0.437 **	1		
7. Openness to experience	0.042	−0.100	−0.196 **	0.082	0.314 **	0.373 **	1	
8. Agreeableness	−0.049	−0.069	−0.209 **	−0.217 **	0.569 **	0.502 **	0.354 **	1
Mean	6.31	3.97	8.33	17.50	31.71	29.37	27.18	29.51
SD	5.958	4.087	7.245	7.824	7.770	7.700	6.465	6.380
Skewness	1.037	0.993	1.003	0.064	−0.614	−0.356	−0.635	−0.956
Kurtosis	0.315	0.408	0.974	−0.502	0.950	0.841	1.203	1.110
Cronbach’s *α*	0.897	0.841	0.831	0.852	0.803	0.721	0.743	0.857

Note: ** *p* < 0.01.

**Table 2:** Predictive role of personality traits on the drive for thinness.

	Model 1				Model 2				Model 3				Model 4				Model 5			
	*B*	*SE*	*β*	*t*	*B*	*SE*	*β*	*t*	*B*	*SE*	*β*	*t*	*B*	*SE*	*β*	*t*	*B*	*SE*	*β*	*t*
N	0.26	0.03	0.35	7.79 **	0.25	0.03	0.34	7.13 **	0.26	0.03	0.34	7.25 **	0.26	0.03	0.34	6.97 **	0.26	0.03	0.34	6.97 **
E					−0.01	0.03	−0.02	−0.48	−0.04	0.04	−0.04	−0.95	−0.04	0.04	−0.05	−0.94	−05	0.04	−0.06	−1.10
C									0.05	0.03	0.06	1.33	0.05	0.04	0.06	1.24	0.04	0.04	0.05	1.01
O													0.01	0.04	0.01	0.09	0.00	0.04	0.00	0.01
A																	0.03	0.05	0.03	0.60
*R* ^2^	0.121				0.122				0.125				0.125				0.125			
Adj *R*^2^	0.120				0.118				0.119				0.117				0.116			
*SE*	5.592				5.597				5.592				5.598				5.602			
*F_(dfn, dfd)_*	60.812 _(1, 441)_				30.467 _(2, 440)_				20.943 _(3, 439)_				15.674 _(4, 438)_				12.592 _(5, 437)_			

Note: ** *p* < 0.01; N = neuroticism; E = extraversion; C = conscientiousness; O = openness to experience; A = agreeableness.

**Table 3:** Predictive roles of personality traits on bulimia.

	Model 1				Model 2				Model 3				Model 4				Model 5			
	*B*	*SE*	*β*	*t*	*B*	*SE*	*β*	*t*	*B*	*SE*	*β*	*t*	*B*	*SE*	*β*	*t*	*B*	*SE*	*β*	*t*
N	0.17	0.02	0.32	7.25 **	0.16	0.02	0.31	6.56 **	0.15	0.02	0.28	6.02 **	0.15	0.02	0.29	6.13 **	0.15	0.02	0.30	6.22 **
E					−0.01	0.02	−0.03	−0.59	0.04	0.02	0.08	1.60	0.05	0.03	0.09	1.84	0.02	0.03	0.03	0.58
C									−0.15	0.02	−0.27	−5.68 **	−0.14	0.02	−0.26	−5.03 **	−0.16	0.02	−0.30	−5.65 **
O													−0.03	0.03	−0.05	−1.18	−0.05	0.03	−0.08	−1.57
A																	0.09	0.02	0.11	1.95
*R^2^*	0.112				0.110				0.173				0.173				0.185			
Adj *R^2^*	0.110				0.108				0.169				0.166				0.176			
*SE*	3.867				3.870				3.739				3.738				3.710			
*F_(dfn, dfd)_*	52.624 _(1, 441)_				26.453 _(2, 440)_				29.666 _(3, 439)_				22.617 _(4, 438)_				19.887 _(5, 437)_			

Note: ** *p* < 0.01; N = neuroticism; E = extraversion; C = conscientiousness; O = openness to experience; A = agreeableness.

**Table 4:** Predictive roles of personality traits on body dissatisfaction.

	Model 1				Model 2				Model 3				Model 4				Model 5			
	*B*	*SE*	*β*	*t*	*B*	*SE*	*β*	*t*	*B*	*SE*	*β*	*t*	*B*	*SE*	*β*	*t*	*B*	*SE*	*β*	*t*
N	0.33	0.04	0.35	7.94 **	0.27	0.04	0.29	6.56 **	0.25	0.04	0.27	5.86 **	0.29	0.04	0.31	6.62 **	0.29	0.04	0.32	6.62 **
E					−0.16	0.04	−0.18	−3.81 **	−0.14	0.04	−0.15	−2.88 **	−0.10	0.04	−0.12	−2.59 **	−0.10	0.05	−0.12	−2.52 **
C									−0.13	0.04	−0.13	−2.77 *	−0.07	0.04	−0.08	−1.59	−0.08	0.05	−0.08	−1.57
O													−0.19	0.05	−0.16	−3.40 **	−0.19	0.05	−0.17	−3.39 **
A																	0.01	0.06	0.01	0.17
*R^2^*	0.125				0.154				0.168				0.189				0.189			
Adj *R^2^*	0.123				0.149				0.162				0.182				0.180			
*SE*	6.785				6.683				6.633				6.554				6.562			
*F_(dfn, dfd)_*	63.030 _(1, 441)_				39.735 _(2, 440)_				29.443 _(3, 439)_				27.518 _(4, 438)_				23.375 _(5, 437)_			

Note: * *p* < 0.05; ** *p* < 0.01; N = neuroticism; E = extraversion; C = conscientiousness; O = openness to experience; A = agreeableness.

## Data Availability

Data may be obtained from the corresponding author.
